# Examining the role of smoking on clinical outcomes after arthroscopic surgery of the hip: a systematic review and meta-analysis

**DOI:** 10.1007/s00590-024-04145-9

**Published:** 2024-12-12

**Authors:** Omkar S. Anaspure, Shiv Patel, Anthony N. Baumann, Theodor Lenz, Nicolas Pascual-Leone, Albert T. Anastasio, Brian C. Lau

**Affiliations:** 1 Perelman School of Medicine, University of Pennsylvania, 3400 Civic Center Blvd, Philadelphia, PA 19140 USA; 2 College of Medicine, Northeast Ohio Medical University, Rootstown, OH USA; 3 Department of Rehabilitation Services, University Hospitals, Cleveland, OH USA; 4 Department Orthopedic Surgery, Hospital for Special Surgery, New York, NY USA; 5 Department Orthopedic Surgery, Duke University, Durham, NC USA

**Keywords:** Smoking, Hip arthroscopy, Total hip arthroplasty, Tobacco, Revision hip arthroscopy, FAIS

## Abstract

**Purpose:**

This study evaluates the impact of smoking on clinical outcomes following hip arthroscopy (HA) through a systematic review and meta-analysis.

**Methods:**

This systematic review and meta-analysis queried PubMed, Scopus, Cochrane, and CINAHL from inception to April 30, 2024, for articles related to smoking and HA outcomes. A random-effects model meta-analysis using relative risk (RR) and 95% confidence intervals was performed to compare smokers and nonsmokers for conversion to total hip arthroplasty (THA) and revision hip arthroscopy (RHA).

**Results:**

Twenty observational studies (*n* = 115,203 patients; 66.95% female; mean age: 36.93 ± 6.53 years; mean follow-up: 22.10 ± 7.56 months) were included. Nine studies investigated smoking and conversion to THA, six examined smoking and RHA, eight assessed smoking and postoperative patient-reported outcomes, and eight evaluated smoking and postoperative complications. Regarding conversion to THA, 5 studies (55.56%) found a significant association, while 4 (44.44%) did not. Meta-analysis from four studies found no significant association between smoking and THA conversion (*p* = 0.48, OR: 1.02; 95% CI: [0.98–1.06]) or smoking and RHA (*p* = 0.305, OR: 1.00; 95% CI: [0.97–1.03]). Only 2 studies (33.33%) found a significant association between smoking and RHA, whereas four did not. Six studies found smoking significantly implicated in complications such as HA failure, increased opioid use, infection risk, and venous thromboembolism (VTE). THA conversion rates were 6.54% (*n* = 14/214) among smokers versus 3.57% (*n* = 13/364) among nonsmokers.

**Conclusion:**

This study found no statistically significant association between smoking and THA conversion, though smokers were observed to experience higher conversion rates overall. Similarly, no significant association was observed for smoking and RHA at 2-year follow-up. However, trends suggest that smokers experience greater risks of adverse outcomes, particularly VTE and HA failure, which should be considered in clinical decision-making.

**Level of Evidence:**

Level III

## Introduction

The adverse effects of smoking have been extensively studied in the context of surgical outcomes, with a large focus on the impact of nicotine and other harmful byproducts produced during the combustion of tobacco [1–4]. Adverse effects of smoking are especially pertinent for patients undergoing orthopedic procedures, as smoking-related complications can result in impaired wound healing, increased rates of osteoporosis, and a higher incidence of fracture nonunion [3, 5–7]. While there is substantial evidence on the impact of smoking on various invasive and complex orthopedic surgeries, only recently has there been a focus on the specific effects of smoking on outcomes following less invasive hip arthroscopy (HA) [2, 8–11].

Femoroacetabular impingement syndrome (FAIS) and acetabular labrum tears are two relatively common hip conditions, affecting approximately 10–15% and 22–55% of the population, respectively. These conditions are often treated surgically using arthroscopic techniques such as acetabuloplasty, microfracture, and labral repair [12–16]. While the poor outcomes of smoking on joint arthroplasty with a focus on perioperative issues such as fracture nonunion, infections, and wound healing complications exists such as those by Yue et al. [22], there are very few comprehensive reviews on the impact of smoking on outcomes after HA for FAIS and associated pathologies [1, 2, 11, 17–22].

The risk of venous thromboembolism (VTE) is an especially important consideration in orthopedic surgery, including HA. While VTE prophylaxis is often emphasized in major orthopedic procedures such as total hip arthroplasty (THA), it is less commonly discussed in the context of HA. Smokers undergoing orthopedic procedures may be at elevated risk for VTE due to the negative effects of nicotine on vascular function and coagulation [23–25]. Current AAOS guidelines recommend VTE prophylaxis based on a risk stratification devised by Parvizi et al. [26] and the VTEstimator iOS app [26]. Though it considers robust risk factors such as hypercoagulability, metastatic cancer, stroke, sepsis, and chronic obstructive pulmonary disease, this model takes and does not take any account for prior smoking history. This further highlights the lack of definitive guidelines available for surgeons to employ when considering the candidacy of patients with extensive smoking history for HA and the question whether the risks of smoking carry over into arthroscopic hip procedures and their severity still remain unanswered.

The most recent systematic review by Emara et al. [2] examined postoperative patient-reported outcomes (PROs) and complications after HA in seven studies [2]. However, it lacked a comprehensive analysis of clinical outcomes, such as revision hip arthroscopy (RHA) and conversion to THA, limiting its conclusions on risk mitigation and surgical decision-making [2]. Additionally, the study did not specifically evaluate the impact of smoking on HA outcomes, highlighting the need for a deeper examination of clinical outcomes beyond PROs. Since Emara et al. [2], numerous cohort studies have explored the relationship between smoking and HA outcomes, warranting an updated systematic review and meta-analysis [8–11, 27–42]. Thus, this study examines the association between smoking and HA outcomes, including revisions and conversion to THA, to better inform preoperative counseling and perioperative care for smokers with hip pathology.

## Methods

### Study creation and initial search

This research is a qualitative systematic review and meta-analysis focusing on the relationship between HA and smoking. The literature search was conducted using PubMed, SCOPUS, Cochrane, and CINAHL from their respective inceptions until April 30th, 2024. The search terms employed included: ("smoking" OR "smoke" OR "smoker" OR "nicotine" OR "tobacco" OR "cigarette" OR "cigar" OR (cigarette smoking[MeSH Terms]) OR (smoking, cigarette[MeSH Terms])) AND ("acetabulum" OR "acetabular" OR "acetabular cartilage" OR "tear" OR "hip surgery" OR "hip arthroplasty" OR "total hip replacement" OR "hip revision" OR "partial hip replacement" OR "hemiarthroplasty" OR "hip arthroscopy" OR "hip osteotomy" OR "resurfacing" OR "hip resurfacing" OR "total hip" OR "hip arthroscopic Surgery"). The study adhered to the latest Preferred Reporting Items for Systematic Reviews and Meta-Analyses (PRIMSA) guidelines for accurate data reporting and was retrospectively registered in the Open Science Framework Registry (10.17605/OSF.IO/36KMF).

### Inclusion and exclusion criteria

The inclusion criteria encompassed retrospective or prospective cohort studies, case series, or randomized controlled trials (RCTs) that involved patients who underwent HA and reported smoking as a demographic variable. Exclusion criteria ruled out studies that were non-arthroscopic, not hip-related, or did not consider smoking as a study variable. Studies deemed to have a high risk of bias, particularly those lacking proper control for confounding variables or incomplete reporting of key outcomes, were excluded to maintain the robustness of the findings.

### Article screening process

Following the execution of the search algorithm across the four databases, all identified articles were uploaded into Rayyan, a public website designed for systematic reviews [43]. An individual screener manually removed duplicates. Two independent reviewers conducted the title and abstract screening, followed by full-text reviews based on the inclusion and exclusion criteria. Additionally, the references of each included article were manually checked for any relevant studies not captured initially. Any disagreements during the screening process were resolved by the first author.

### Data extraction

Two authors carried out the data extraction. Extracted data included the first author's name, year of publication, type of procedure, number of patients, gender, average age, follow-up duration, conversion to THA, significant findings related to smoking and HA, and other relevant qualitative data with associated p-values for narrative reporting.

### Study variable definition

The term "smoking" was defined to include any patients who currently or previously used tobacco, nicotine, chewing tobacco, or nicotine products, as noted in the included studies [8–10, 36]. This standardization aimed to ensure consistent patient classification for meta-analysis.

### Article quality grading

All observational studies in this systematic review were categorized as either “comparative” or “non-comparative” and assessed for quality using the Methodological Index for Non-Randomized Studies (MINORS) scale [44]. Comparative studies were scored out of 24 points, while non-comparative studies were scored out of 16 points. The scale consists of eight items for comparative studies and 12 items for non-comparative studies, with each item rated from 0 to 2 points. Articles were classified as “high-quality”, “moderate-quality”, or “low-quality” based on their scores. High-quality comparative studies scored 24 points, moderate-quality scored 15–23 points, and low-quality scored less than 15 points. For non-comparative studies, high-quality scored 16 points, moderate-quality scored 10–15 points, and low-quality scored less than 10 points [45]. Studies with a high risk of bias (low-quality based on MINORS scores) were excluded from the meta-analysis to ensure that only moderate- and high-quality studies were included in the final synthesis.

### Statistical analysis

Statistical analysis was performed using the Statistical Package for the Social Sciences (SPSS) version 29.0 (Armonk, NY: IBM Corp). Frequency-weighted means and other descriptive statistics were used where no statistical significance could be calculated. A random-effects model was chosen for the meta-analysis to account for the inherent variability between studies (e.g., differences in study populations, methodologies, or sample sizes), ensuring that the results reflect both within-study and between-study variation. This approach was used to provide more generalized conclusions, given the heterogeneity expected among observational studies. The random-effects binary outcomes meta-analysis was conducted using relative risk (RR) and 95% confidence intervals (CIs) to compare binary outcomes across studies. Instances of zero complications were adjusted to 0.5 in binary outcomes, as noted in other literature; this adjustment was applied to one study [10, 46]. A forest plot was created to illustrate the relationships between variables.

## Results

### Initial search results

**Fig. 1:**
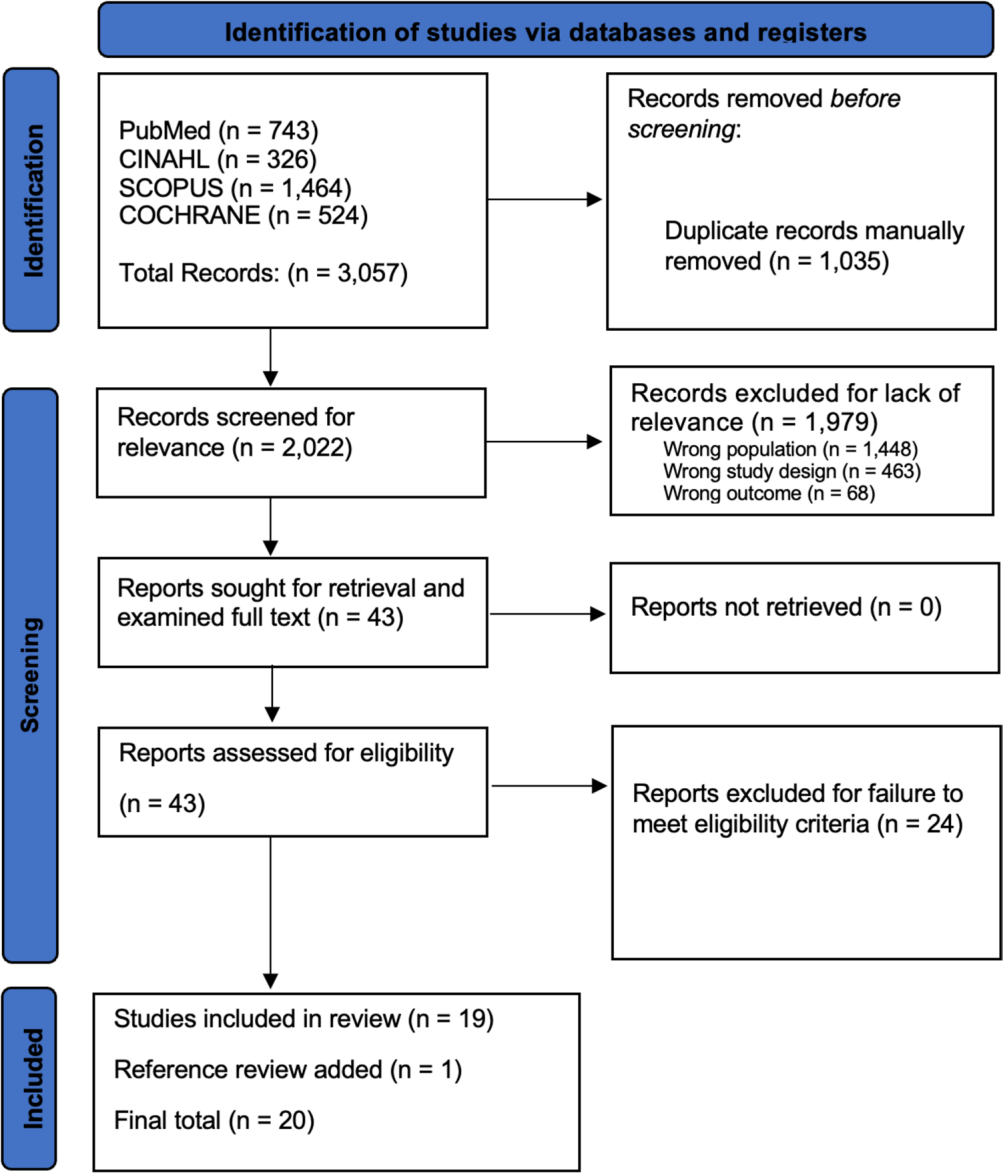
The Preferred Reporting Items for Systematic Reviews and Meta-Analyses (PRISMA) diagram outlining the entire search progress, from initial search in four databases to final article inclusion

The database search initially identified 3,057 articles. After automated deduplication, 2,022 articles remained. Following title and abstract screening, 43 articles were selected for full-text analysis. A citation search yielded one additional study. Ultimately, 20 articles met the inclusion criteria and were included in the data extraction process [8–11, 27–42] (Fig. 1).

### Article quality results

All 20 articles were observational studies, consisting of 15 retrospective and five prospective studies, and were assessed using the MINORS scale. Among these, there were two non-comparative studies and 18 comparative studies. The non-comparative studies (*n* = 2) had a mean MINORS score of 10.0 out of 16 points. The comparative studies (*n* = 18) had a MINORS score of 17.2 ± 2.4 out of 24 points. Ultimately, 19 articles were classified as "moderate-quality," one article was "low-quality," and none were rated as "high-quality" (Table 1).

**Table 1:** The methodological index for non-randomized studies (MINORS) grading for all included articles in this systematic review

First author (Year)	Study type	Total MINORS score	Clearly stated aim	Inclusion of consecutive patients	Prospective collection of data	End points appropriate to study aim	Unbiased assessment of study end point	What follow-up period appropriate to study aim	Less than 5% lost to follow-up	Prospective calculation of the study size	Adequate control group	Contemporary groups	Baseline equivalence of groups	Adequate statistical analysis
Tiao et al. [40]	Comparative	18	2	2	0	2	0	2	2	0	2	2	2	2
Lee et al. [9]	Comparative	18	2	2	0	2	0	2	0	2	2	2	2	2
Niknam et al. [39]	Comparative	16	2	2	0	2	0	2	1	0	2	2	1	2
Kunze et al. [35]	Comparative	14	2	2	0	2	0	2	0	0	2	2	0	2
Lynch et al. [37]	Comparative	16	2	2	2	2	0	2	0	0	2	2	0	2
Jimenez-Owens et al. [10]	Comparative	20	2	2	0	2	0	2	2	2	2	2	2	2
Jimenez-George et al. [8]	Comparative	20	2	2	0	2	0	2	2	2	2	2	2	2
Lall et al. [36]	Comparative	20	2	2	2	2	0	2	0	2	2	2	2	2
Kamath et al. [32]	Non-comparative	10	2	2	0	2	0	2	0	2	–	–	–	–
Beck et al. [29]	Non-comparative	10	2	2	2	2	0	2	0	0	–	–	–	–
Cancienne et al. [11]	Comparative	18	2	2	2	2	0	2	0	2	2	2	0	2
Khazi et al. [34]	Comparative	16	2	2	0	2	0	2	2	0	2	2	0	2
Holler et al. [31]	Comparative	15	2	2	0	2	0	2	1	0	2	2	0	2
Berlinberg et al. [30]	Comparative	20	2	2	0	2	0	2	2	2	2	2	2	2
Kester et al. [33]	Comparative	16	2	2	0	2	0	2	2	0	2	2	0	2
Baron et al. [28]	Comparative	14	2	2	0	2	0	2	0	0	2	2	0	2
Wang et al. [41]	Comparative	16	2	2	0	2	0	2	1	0	2	2	1	2
Yao et al. [42]	Comparative	16	2	2	0	2	0	2	0	2	2	2	0	2
Mohtadi et al. [38]	Comparative	22	2	2	2	2	0	2	2	2	2	2	2	2
Anciano Granadillo et al. [27]	Comparative	15	2	2	0	2	0	2	1	0	2	2	0	2

### Patient and study characteristics

In total, 115,203 patients (frequency weighted mean age: 36.93 ± 6.53 years; 66.95% female) were included in this review, and all patients underwent HA as the primary method of correcting the underlying pathology. The most frequent diagnosis was FAIS reported directly by ten studies (*n* = 29,060) followed by labral tear acetabular labrum tear reported by five studies (*n* = 13,117). The mean postoperative follow-up time across the 16 studies that reported this metric was 22.10 ± 7.56 months. All studies were conducted in the USA. Further patient and study characteristics can be found in Table 2, and significant study findings can be found in Table 3.

**Table 2:** Study demographics table with relevant study characteristics such as diagnosis, procedure type, mean age, and mean follow-up time

Author (Year)	Study Type	Diagnosis	Type of procedure	Smoker/Nonsmoker	Patients	Age	Male/female	Follow-up time
Tiao [40]	Retrospective case control	FAIS, Labral tear	Femoroacetabular osteoplasty, isolated debridement, isolated labral repair	Smokers	147	41.16	–	2 years
				Nonsmokers	4901			
Lee [9]	Retrospective cohort	FAIS	Labral repair, labral reconstruction, labral debridement, capsular repair, femoroplasty, acetabular microfracture, femoral head microfracture	Former smokers	84	45.0 ± 13.5	Male (*n* = 22) Female (*n* = 62)	38.6 [27.5–48.2] mo
				Nonsmokers	84	45.9 ± 14.1	Male (*n* = 26) Female (*n* = 58)	39.0 [28.3–48.1] mo
Niknam [39]	Retrospective cohort	FAIS	–	Smokers	14,677	38.9	Male (*n* = 4237) Female (*n* = 10,440)	2 years
				Nonsmokers				
Kunze [35]	Retrospective case control	FAIS	–	Smokers	184	30.2	Male (*n* = 707) Female (*n* = 1406)	2 years
				Nonsmokers	1528			
Lynch [37]	Prospective cohort	FAIS, Labral tear	Labral repair, labral debridement, other labral treatment, acetabuloplasty, femoroplasty	Smokers	43	33 (median)	Male (*n* = 97) Female (n-288)	1 year
				Nonsmokers	274			
Jimenez-Lee-Owens [10]	Retrospective cohort	FAIS	Labral reconstruction, labral repair, selective labral debridement, acetabuloplasty, femoral osteoplasty, acetabular microfracture, femoral head microfracture	Smokers	35	39.4 ± 13.0	–	64.6 ± 4.1 mo
				Nonsmokers	70	38.1 ± 15.2		67.3 ± 10.4 mo
Jimenez-Lee-George [8]	Retrospective cohort	Labral tear	Capsular repair, femoroplasty, acetabular microfracture, femoral head microfracture	Smokers	20	41.4 ± 10.4	–	39.9 ± 13.0 mo
				Nonsmokers	60	42.5 ± 10.1		35.0 ± 10.8 mo
Lall [36]	Prospective cohort	FAIS	Labral reconstruction, labral repair, labral debridement, acetabular microfracture, capsular release, capsular plication, ligamentum teres debridement, isolated/combined femoroplasty, isolated/ combined acetabuloplasty, iliopsoas fractional lengthening, synovectomy, notchplasty	Smokers	75	41.7 ± 11.1 (18.8–66.9)	Male (*n* = 42) Female (*n* = 33)	42.5 ± 18.6 (24.0–88.6) mo
				Nonsmokers	150	41.7 ± 11.1 (18.9–68.7)	Male (*n* = 84) Female (*n* = 66)	47.6 ± 19.5 (24.0–96.1) mo
Kamath [32]	Retrospective case series	Labral pathology	Labral repair, labral debridement	Smokers	8	42	–	58 mo
				Nonsmokers	44			
Beck [29]	Prospective case series	FAIS	–	Smokers	97	33.5 ± 12.6	Male (*n* = 314) Female (*n* = 662)	24 mo
				Nonsmokers	879			
Cancienne [11]	Prospective cohort	FAIS	Labral Repair, acetabular rim trimming, femoral osteochondroplasty, capsular plication, trochanteric bursectomy, synovectomy	Smokers	40	35.3 ± 6.4	Male (*n* = 16) Female (*n* = 24)	33.7 ± 3.1 mo
				Nonsmokers	1062	31.6 ± 12.3	Male (*n* = 364) Female (*n* = 698)	
Khazi [34]	Retrospective cohort	Nonemergency	diagnostic arthroscopy, chondroplasty, removal of loose or foreign body, synovectomy, femoroplasty, acetabuloplasty, labral repair	Smokers	967	–	Male (*n* = 4389) Female (*n* = 5088)	–
				Nonsmokers	8510			
Holler [31]	Retrospective cohort	–	diagnostic arthroscopy, chondroplasty, removal of loose or foreign body, synovectomy, femoroplasty, acetabuloplasty, labral repair	Smokers	6137	–	Male (*n* = 18,530) Female (*n* = 41,631)	–
				Nonsmokers	54,024			
Berlinberg [30]	Retrospective cohort	–	diagnostic arthroscopy, chondroplasty, removal of loose or foreign body, synovectomy, femoroplasty, acetabuloplasty, labral repair	Smokers	415	34.86	Male (*n* = 544) Female (*n* = 1644)	At least within 90 days
				Nonsmokers	1773			
Kester [33]	Retrospective case control	–	Labral repair, pincer resection, cam resection, chondroplasty, synovectomy, loose body removal	Smokers	3,957	35.8 ± 13.1	Male (*n* = 1794) Female (*n* = 2163)	Minimum of 2 years
				Nonsmokers				
Baron [28]	Retrospective case control	–	Most common was labral resection, others included diagnostic arthroscopy, chondroplasty, "removal of loose or foreign body", synovectomy, femoroplasty, and acetabuloplasty	Smokers	785	–	Male (*n* = 282) Female (*n* = 503)	Minimum of 1 year
				Nonsmokers				
Anciano Granadillo [27]	Retrospective cohort	–	–	Smokers	297	–	Male (*n* = 619) Female (*n* = 1089)	Minimum 6 months
				Nonsmokers	1411			
Wang [41]	Retrospective cohort	Nonemergency	diagnostic arthroscopy, removal of foreign body, labral debridement, synovectomy, femoroplasty, acetabuloplasty, labral repair	Smokers	1838	–	Male (*n* = 2796) Female (*n* = 4824)	–
				Nonsmokers	5782			
Yao [42]	Retrospective case control	FAIS	–	Smokers	4,730	38.15940803	–	2 years
				Nonsmokers				
Mohtadi [38]	Prospective cohort	–	–	Smokers	15	34.9 ± 10.3	Male (*n* = 55) Female (*n* = 60)	3 mo
				Nonsmokers	100

**Table 3:** Key narrative findings from each study such as conversion to THA, RHA, and PROs

Author (Year)	Key findings
Tiao [40]	Tobacco use was not significantly associated with conversion to THA (OR: 1.56 [0.93-2.62], *p* = 0.091).
Lee [9]	Former smokers achieved significantly higher patient satisfaction at 2-years than did nonsmokers (9.0 vs 8.0, respectively; *P* = .031). Postoperative PROs were similar between groups. No difference in RHA or conversion to THA between groups.
Niknam [39]	Tobacco use (OR:1.24, 95%CI: 1.03–1.49, *p* = 0.022) was significantly associated with higher odds of 2-year RHA and conversion to THA (OR:1.24, 95%CI: 1.03–1.49, *p* = 0.022), (OR:1.68, 95%CI: 1.38–2.04, p < 0.001), respectively.
Kunze [35]	Smoking significantly increased the risk for loss to follow-up after HA (OR: 1.07 [1.04-1.12], *p* = 0.021).
Lynch [37]	Current /former smokers had significantly worse PRO scores than nonsmokers [(OR: 0.44 [0.24-0.82], *p* = 0.01); (OR: 2.07 [1.13-3.79], *p* = 0.018); (OR: 0.42 [0.22-0.78], *p* = 0.006)]
Jimenez-Lee-Owens [10]	No significant difference in minimum 5-year PROs or improvement in PROs between groups. No significant difference in RHA or conversion to THA between groups.
Jimenez-Lee-George [8]	No significant difference in minimum 2-year PROs improvement between groups. There was no significant difference in RHA between groups. There was a significant difference in conversion to THA (4 vs 1, *p* = 0.013). There was a significant difference in the total revision rate between smokers and nonsmokers (5 vs 3, *p* = 0.031).
Lall [36]	Significant difference found in only postoperative scores for the mHHS (*p *< 0.05), NAHS (*p *< 0.05), and IHOT-12 (*p *< 0.05) between the smokers and nonsmokers. No significant difference in RHA or conversion to THA between groups. No difference in time to revision or THA between groups
Kamath [32]	Smoking was found to be a significant negative predictor of good or excellent outcomes by mHHS (OR: 0.082 [0.009-0.725], *p* = 0.023) and was not significantly associated with return to activity (OR: 0.257 [0.054-1.233], *p* = 0.089).
Beck [29]	Nonsmoking is a preoperative predictor for achieving the VAS pain score threshold for significant clinical benefit (OR, 0.078 [0.007-0.915], *p* = 0.042).
Cancienne [11]	Smokers had worse postoperative PRO scores compared to nonsmokers (*p *< 05)
Khazi [34]	Smoking is a significant risk factor for VTE after HA (OR 1.26, 95% CI 1.04-1.53, *P* = 0.0177). Those with VTE after HA had higher incidence of smokers than those without VTE (16.67% vs 10.21%; P ¼ = .0282).
Holler [31]	Smoking was not significantly associated with VTE after HA. Smoking was associated with decreased thromboprophylaxis use (OR 0.80; 95% CI, 0.65-0.98) but was not associated with any DVT or PE within 90 days (aOR 1.16; 95% CI, 0.85-1.59).
Berlinberg [30]	Patients undergoing HA were more likely to use tobacco (176 inpatients [21.2%] vs 239 outpatients [17.6%], *P* = .05). Tobacco use was not a significant risk for complication within 90 days of HA.
Kester [33]	Tobacco use was independently associated with significantly higher rate of conversion to THA (OR 1.9, 95% CI: 1.2-3.2, *p* < 0.001)
Baron [28]	Smoking was both a risk factor and independent variable associated with conversion to THA after HA [(OR 2.61, 95% CI [1.46-4.52], *p* < 0.01); (OR 2.05, [1.68-1.88], *p* = 0.02)] and for HA failure (OR 1.94 95% CI 1.16-3.16, *p* < 0.01).
Anciano Granadillo [27]	Tobacco use was found to be a significant risk factor for prolonged opioid use after HA (OR: 1.63, *p* < 0.0001).
Wang [41]	Tobacco use was associated with increased infection risk, independently of preoperative hip injection for private and Medicare patients (private: OR 2.1, 95% CI 1.2-3.6, *P* = 0.006; Medicare: OR 1.7, 95% CI 1.0-2.8, *P* = .033)
Yao [42]	Smokers were more likely than nonsmokers to convert to THA (6% vs 3%, *p* = 0.04). When the cohort size was increased to 11,323 by lifting 2-year follow-up requirement, the hazards ratio for smoking and conversion to THA was significant (HR: 1.55 (1.14 to 2.11), *p* = 0.005)
Mohtadi [38]	Smoking was a significant risk factor for the onset of thromboembolic events after HA

### The impact of smoking on conversion to total hip arthroplasty

**Fig. 2:**
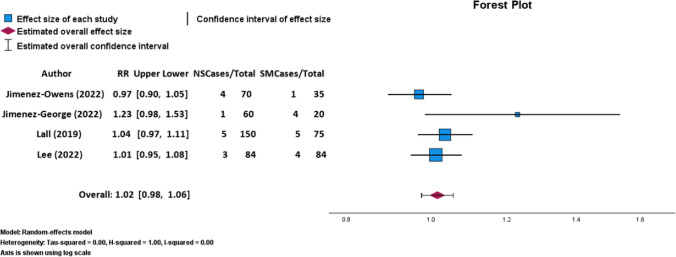
Random-effects model forest plot demonstrating relationship between conversion to THA and smoking status for four studies [8–10, 36]. Effect measures used were RRs and CIs. Abbreviations: SM, smokers; NS, nonsmokers; RR, risk ratio; CI, confidence interval. Heterogeneity: Tau-squared = 0.00, H-squared = 1.00, I-squared = 0.60. Axis is shown using log scale

Of the 20 included studies, nine studies reported outcomes regarding smoking and conversion to THA. Five studies (55.56%) found smoking to be significantly associated with conversion to THA and 4 (44.44%) did not [8–10, 28, 33, 36, 39, 40, 42]. THA conversion rates were 6.54% (*n* = 14/214) among smokers versus 3.57% (*n* = 13/364) among nonsmokers. Niknam et al. [39] (*n* = 14,677) identified tobacco use as a significant predictor for both revision HA and conversion to THA in patients treated with HA for FAIS (OR: 1.68, 95% CI: 1.38–2.04, p < 0.001) [39]. Similar to Niknam et al. [39], three additional studies [8, 33, 42] looking at a cumulative 8767 patients undergoing HA for FAIS or labral tears all found the rate of conversion to THA after HA significantly higher in the smoking group versus the never-smoking group (*p* = 0.013 [8]; OR 1.9, 95% CI: 1.2–3.2, *p* < 0.001 [33]; 6% vs 3%, *p* = 0.04 [42], respectively). Baron et al. [28] reinforced these findings by demonstrating that smoking was both a risk factor and an independent variable linked to conversion to THA after HA in a study involving 785 patients who underwent HA [(OR 2.61, 95% CI [1.46–4.52], p < 0.01); (OR 2.05, [1.68–1.88], *p* = 0.02), respectively] [28]. However, the remaining four studies (*n* = 5,546 patients total) found that there was no significant relationship between smoking and conversion to THA [9, 10, 36, 40]. When examining only the four studies that reported the number of conversions to THA stratified by smoking status via a random-effects meta-analysis, we found that there was not a statistically significant association between smoking status and conversion to THA (*p* > 0.05; RR: 1.02; 95% CI: 0.98, 1.06; Table 4; Fig. 2).

**Table 4:** Conversion to total hip arthroplasty after hip arthroscopy stratified by smoking status

Author	Smoker/nonsmoker	# of patients	Conversion to THA (4)
Lee [9]	Smokers	84	4 (4.8%)
	Nonsmokers	84	3 (3.6%)
Jimenez-Lee-Owens [10]	Smokers	35	1 (2.9%)
	Nonsmokers	70	4 (5.7%)
Jimenez-Lee-George [8]	Smokers	20	4 (20.0%)
	Nonsmokers	60	1 (1.7%)
Lall [36]	Smokers	75	5 (6.7%)
	Nonsmokers	150	5 (3.3%)

### The impact of smoking on revision hip arthroscopy

Of the 20 included studies, six studies reported outcomes regarding the impact of smoking on RHA. Two studies (33.33%) reported a significant association between smoking and RHA and 4 studies (66.67%) reported no significant association. Jimenez-George et al. [8] conducted a study with 20 smokers and 60 nonsmokers, finding that the revision rate was notably higher among smokers compared to nonsmokers, with significant p-values of 0.013 and 0.031, respectively [8]. Similarly, Niknam et al. (*n* = 14,677) found that smoking was significantly associated with higher odds of 2-year RHA (OR:1.24, 95%CI: 1.03–1.49, *p* = 0.022). The remaining studies, however, (*n* = 1,283 patients total) found no such associations [9, 10, 28, 36]. Additionally, a second meta-analysis was performed for four studies which reported the number of conversions to RHA stratified by smoking status. We found that there was not a statistically significant association between smoking status and conversion to THA (p > 0.05; RR: 1.00; 95% CI: [0.97, 1.03]; tau-squared = 0.00; H-squared = 1.00; and I-squared = 0.00) [8–10, 36].

### The impact of smoking on postoperative complications

Eight studies (40%) of the 20 included studies reported postoperative complications based on patient smoking status [27, 28, 30, 31, 34, 35, 38, 41]. Holler et al. [31] conducted a study with 60,161 patients and found no significant difference in the incidence of deep vein thrombosis or pulmonary embolism (DVT/PE) between smokers and nonsmokers during the immediate 90-day follow-up period (OR 1.16; 95% CI, 0.85–1.59) [31]. In contrast, Khazi et al. [34] and Mohtadi et al. [38] identified smoking as a significant risk factor for VTE beyond 90-day post-HA, with Khazi et al. reporting an odds ratio of 1.26 (95% CI 1.04–1.53, *p* = 0.018) and Mohtadi et al. also finding a significant association (*p* < 0.05) [34, 38]. Additionally, four studies (20%) found that smoking was also associated with complications such as HA failure (OR 1.94 95% CI 1.16–3.16, *p* < 0.01), opioid use (OR: 1.63, *p* < 0.0001), increased infection risk (*p* < 0.05), and greater risk for loss to follow-up after HA (OR: 1.07 [1.04–1.12], *p* = 0.021) compared to nonsmokers, respectively [27, 28, 35, 41].

### The impact of smoking on postoperative patient-reported outcomes

Of the 20 included studies, eight studies described postoperative PRO scores (Table 5) [8–11, 29, 32, 36, 37]. The most frequently reported measures were the modified Harris Hip Score (mHHS), the visual analog scale (VAS) for pain, the Hip Outcome Score–Sports Specific (HOS-SS), the Hip Outcome Score–Activities of Daily Living (HOS-ADL), and the VAS for satisfaction. Collectively, the eight studies comprised 3025 hips (smokers, *n* = 402; nonsmokers, *n* = 2623).

**Table 5:** Comprehensive postoperative PRO scores and improvement from baseline for eight studies

	Postoperative			Improvement			MCID achievement rates			PASS achievement rates		
PRO	Smoker	Nonsmoker	*p*	Smoker	Nonsmoker	*p*	Smoker	Nonsmoker	*p*	Smoker	Nonsmoker	*p*
Lee [9]												
HOS-SS	65.6 [45.7–91.1]	64.3 [37.5–100]	0.705									
VAS for pain	2.0 [0.5–4.0]	1.5 [0.0–4.0]	0.300	4.0 [1.5–6.0]	3.8 [2.0–9.0]	0.532	81.60%	87.00%	0.483			
mHHS	86.0 [68.0–96.0]	86.0 [65.0–96.0]	0.648	22.6 ± 18.8 (− 15.8–75.0)	21.9 ± 22.2 (− 33.0–68.0)	0.963	75%	78.90%	0.723			
NAHS	86.3 [76.6–92.8]	85.6 [72.2–97.5]	0.730	21.6 ± 18.0 (− 41.5–60.0)	24.5 ± 22.1 (− 40.5–79.0)	0.392	77.50%	81.90%	0.647			
IHOT-12	71.3 [41.7–83.8]	69.9 [41.2–89.0]	0.855									
Patient satisfaction	9	8	-									
Jimenez-Owens [10]												
HOS-SS	70.4 ± 30.4 (5.6 to 100)	81.9 ± 22.0 (27.8 to 100)	0.076	40.3 ± 36.5 (− 50.0 to 91.7)	34.5 ± 30.1 (− 61.1 to 100)	0.458						
mHHS	81.3 ± 19.7 (40.0 to 100)	88.0 ± 13.2 (45.0 to 100)	0.647	27.5.4 ± 24.7 (− 32.3 to 66.0)	21.8 ± 19.8 (− 26.0 to 64.0)	0.108				79.40%	74.20%	0.744
NAHS	78.8 ± 24.1 (32.5 to 100)	87.6 ± 12.6 (46.3 to 100)	0.351	30.6 ± 22.6 (− 27.5 to 73.8)	23.9 ± 21.8 (− 33.0 to 80.8)	0.155				64.70%	77.30%	0.27
IHOT-12	74.7 ± 24.2 (2.2 to 100)	82.2 ± 18.3 (25.8 to 100)	0.122							50.00%	68.20%	0.12
Jimenez-George [8]												
VAS for pain	1.9 ± 2.2 (0.0 to 7.0)	2.3 ± 2.5 (0.0 to 9.0)	0.701	1.9 ± 2.2 (0.0 to 7.0)	2.3 ± 2.5 (0.0 to 9.0)	0.701	70.00%	80.00%	0.536			
mHHS	84.8 ± 16.5 (59.0 to 100)	86.5 ± 16.6 (35.0 to 100)	0.654	31.7 ± 22.7 (− 9.0 to 76.0)	20.1 ± 22.6 (− 76.0 to 64.0)	0.100	70.00%	76.70%	0.766	55.00%	75.00%	0.159
NAHS	83.2 ± 13.5 (56.3 to 100)	85.1 ± 17.8 (27.5 to 100)	0.294	26.5 ± 19.4 (− 8.8 to 60.0)	18.8 ± 18.3 (− 40.5 to 51.3)	0.193	65.00%	75.00%	0.563	40.00%	61.70%	0.152
Patient satisfaction	8.5 ± 1.8 (4.0 to 10.0)	8.1 ± 2.3 (0.0 to 10.0)	0.498									
Lall [36]												
HOS-SS	60.8 ± 28.8	69.6 ± 26.7	0.08	30.3 ± 28.5	28.1 ± 31.6	0.69						
mHHS	76.3 ± 20.5	83.4 ± 16.1	< 0.05	20.8 ± 21.3	20.4 ± 20.0	0.10						
VAS for pain	3.1 ± 2.7	2.3 ± 2.2	0.14	− 2.7 ± 3.0	− 3.0 ± 2.8	0.29						
NAHS	77.0 ± 19.9	83.5 ± 16.8	< 0.05	24.5 ± 19.2	22.7 ± 19.4	0.63						
IHOT-12	62.5 ± 26.7	73.1 ± 25.0	< 0.05									
Patient satisfaction	7.9 ± 2.2	8.0 ± 2.5	0.87									
Cancienne [11]												
HOS-ADL	80.4 ± 19.9	89.1 ± 11.4	0.013				64.60%	77.80%	< 0.001	55.10%	67.80%	< 0.001
HOS-SS	65.8 ± 17.1	75.6 ± 14.1	0.046				80.80%	85.70%	0.078	69.20%	62.40%	0.14
VAS for pain	74.5 ± 21.8	80.3 ± 14.2	0.011									
mHHS	3.2 ± 2.9	1.8 ± 2.2	0.14				72.20%	83.40%	< 0.001	65.20%	74.60%	< 0.001
VAS for satisfaction	74.3 ± 29.0	81.8 ± 22.6	0.14									
Lynch [37]												
HOOS-pain			OR: 0.44 [0.24–0.82], *p* = 0.01									
HOOS-PS			OR: 2.07 [1.13–3.79], *p* = 0.018)									
VR-12 MCS			OR: 0.42 [0.22–0.78], *p* = 0.006									
Kamath [32]												
mHHS			OR: 0.082 [0.009–0.725], *p* = 0.023									
Beck [29]												
VAS for pain			OR, 0.078 [0.007–0.915], *p* = 0.042									

Hip Outcome Score, Score Sport (HOS-SS); Hip Outcome Score, Activity of Daily Living (HOS-ADL); Physical Function Shortform of the Hip Disability and Osteoarthritis Outcome Score (HOOS-PS); Hip Disability and Osteoarthritis Outcome Score, pain (HOOS-pain); Visual Analog Scale (VAS); Non Arthritic Hip Score (NAHS); International Hip Outcome Tool 12 (IHOT-12); Veterans Rand 12 Mental Component Score (VR-12 MCS); modified Harris Hip Score (mHHS)

## Discussion

This study investigated the impact of smoking on clinical outcomes after HA, providing a summarized examination of this important and underexamined topic. As smoking has been widely demonstrated to increase the risk for surgical site infections, wound healing issues, and prolonged recovery times in orthopedic patients, it is critical to examine the impact of smoking in relation to clinical outcomes after HA [1–4]. This systematic review showed mixed findings regarding the significance of smoking significance in conversion to THA. Our meta-analysis showed that no significant association found in the pooled data regarding the influence of smoking and conversion to THA or RHA, respectively. However, individual studies showed a trend of finding no association between smoking and RHA, unlike THA. Qualitatively, smoking was also associated with higher incidences of HA failure, infection risk, and thromboembolic events.

Over the past decade, HA has become more common, with an almost 250% increase between 2007 and 2011 in the USA, with similar trends seen globally [47–50]. However, despite advancements in imaging and injury detection, the procedure remains associated with complications such as mechanical instability, adhesions, and progression to arthritis or femoral head necrosis, especially in smokers who may have compromised healing capacities [51]. Patients experiencing complications like postoperative arthritis or femoral head necrosis often require conversion to THA [48, 52]. Smokers sustain high levels of circulating carbon monoxide and have reduced oxidative metabolism required for adequate cell proliferation to address injury as well as reduced collagen I production [2, 18, 53]. Thus, smokers are likely at higher risk of such progression and may experience poor outcomes following surgery. Emara et al. [2] were unable to establish a relationship between smoking and conversion to THA in their systematic review due to a lack of sufficient sample size across the included studies, but discussed observing higher complications seen in the literature such as need for conversion to THA among patients who undergoing hip and knee arthroplasty [2, 54]. The current study improved upon this previous systematic review by identifying additional cohort studies reporting a significant impact of smoking on conversion to THA in HA, with larger sample sizes than the four studies reporting contradictory findings. However, the pooled meta-analysis in this study found no conclusive relationship between smoking and conversion to THA. This finding should be interpreted cautiously due to the limited number of patients in the analysis and mixed methods of the pooled studies. Newer, larger sample size studies with more robust methodologies and demographic reporting may have more appropriately identified the relationship between smoking and complications following HA compared with earlier literature.

Taken together, the literature suggests that the unequivocal results regarding the role of smoking on complications following HA may be due to a younger demographic undergoing HA compared to other orthopedic procedures where smoking portends a poor prognosis [2]. Our study reports a mean age of 36.93 years, as compared to much older mean ages of patients who received total hip or knee arthroplasty (67.7 and 69.9 years at surgery, respectively) [2, 55, 56]. Age at the time of surgery is an important factor when evaluating the impact of smoking, as the harmful effects of long-term tobacco use are amplified over the years [57]. This is compounded by the higher prevalence of comorbid factors such as elevated BMI, previous complications from surgeries, and medical history, particularly in older patients who smoke [57]. The relatively young population of patients undergoing HA may partially explain the absence of obvious elevation in need for conversion to THA among the pooled group of studies at 2-year follow-up. However, it is important to recognize that this finding does not indicate that smoking does not have a detrimental effect on the outcomes following HA. Longer-term follow-up may reveal not only higher rates of conversion to THA in cohorts that are smoking but may also further delineate more rapid onset of functional impairment, poor rates of return to activity and sport, and various other negative outcomes associated with smoking. Thus, further large cohort studies are needed to determine whether smoking is an independent variable associated with poor HA outcomes.

Another common procedure performed after HA is RHA, performed primarily to address symptoms of residual cam- or pincer-type deformity that was either unaddressed or under-resected during the initial HA procedure [58]. Patients experiencing pain or poor functional status might be considered for repeat procedures, especially if postoperative advanced imaging reveals missed pathology. The time to RHA after an initial HA is typically shorter than the time to THA, averaging 2 years compared to three years [48, 58]. This finding may be due to symptoms and complications from incomplete resection or deformity being experienced sooner than the arthritic change leading to THA. Alternatively, surgeons may be reticent to offer THA in this younger patient and may more quickly proceed to RHA. In this study, a comparison of smokers and nonsmokers showed a strong trend of no significant association between smoking and RHA at 2 years post-HA, supported by pooled analysis. In contrast, multiple cohort studies reported a significant association between smoking and time to THA at the same time point, with an average follow-up of 22.10 months. These findings may be considered intuitive, as RHA may result more commonly due to interoperative technique errors, while need for conversion to THA may depend substantially on patient-specific biologic considerations (such as smoking). Thus, these findings lend an element of veracity to the results of the present metanalysis.

Smoking is also widely linked to various adverse postoperative complications. Emara et al. [2] reported a significant association between smoking and thromboembolism events in their review of seven cohort studies. Our study describes a similar trend, with higher rates of VTE in smokers following HA compared to nonsmokers. We also observed increased risks of HA failure, postoperative infection, and opioid use in smokers. Our study reports a reduced quality of life for patients who smoke undergoing HA, often experiencing more pain and discomfort than nonsmokers. The causative factors behind these findings are likely multifactorial. Negative effects of smoking on secondary pathologies such as neuropathy have been well documented, with smoking patients expected to experience increasing intensity of nerve pain based on their duration of smoking history [59]. As a result, smokers often experience higher acute pain after surgery and consume more opioids as a mechanism to combat discomfort [60]. VTE risk is also far higher in smokers undergoing various forms of surgery. While the risk of VTE following arthroscopic surgery remains low across the literature, the actual incidence may be confounded by the low rate of reporting in arthroscopic studies [23, 25]. Nevertheless, smoking continues to be a significant risk factor for VTE, as it reduces tissue blood flow, causes endothelial dysfunction, raises plasma fibrinogen levels, and disrupts the coagulation-fibrinolysis cascade at multiple levels [24, 34, 61–63]. Such factors can strongly contribute to VTE propagation following surgery, especially in patients with largely sedentary lifestyle habits.

Similar findings of smoking have been reported in other arthroscopic procedures of the shoulder and knee. Patients who smoke have lower American Shoulder and Elbow Surgeons (ASES) scores than nonsmokers at 2 years following rotator cuff repair [2, 64]. Moreover, patients who smoke tended to have larger tears at initial presentation [64]. This suggests that smoking may contribute to more severe initial injury, potentially due to nicotine's impact on collagen synthesis and overall tissue quality, which impairs the body's ability to respond to mechanical stress and injury. The fact that smokers present with more advanced pathology highlights the importance of addressing smoking cessation preoperatively to mitigate these effects. Similarly, Anaspure et al. [20] observed trends of higher rates of failure after meniscus repair in patients who smoke compared to nonsmokers [20]. This aligns with other findings, suggesting that smoking exacerbates poor healing environments, possibly due to decreased blood flow, hypoxia, and inflammatory responses. The consistently higher failure rates seen across various joint repair procedures in smokers underscore the systemic nature of smoking-related complications, not limited to one specific joint but extending across musculoskeletal systems. Both Berglund et al. and Heyer et al. reported that in patients undergoing arthroscopic knee meniscectomy, meniscal repair, chondroplasty, synovectomy, anterior cruciate ligament repair, or shoulder decompression, smoking was associated with higher VAS pain scores postoperatively and a higher overall complication rate [2, 17, 65]. This points to a potential multifactorial cause where smoking not only impairs healing but also amplifies postoperative pain responses, possibly due to chronic nicotine-induced neuropathy or reduced vascular supply, both of which may prolong recovery. Understanding this relationship between smoking and postoperative pain should prompt clinicians to implement more targeted pain management and smoking cessation strategies. Naimark et al. [64] also noted smokers to present with larger glenoid labral tears and worse function recovery after rotator cuff repair compared to nonsmokers [64]. This further supports the hypothesis that smoking may cause progressive tissue degeneration, likely due to nicotine's inhibition of cellular repair mechanisms. These findings collectively suggest that smoking not only delays recovery but may actively worsen the initial severity of injury and postoperative outcomes. This should be considered when planning treatment protocols, as smokers may benefit from more aggressive preoperative interventions, such as nicotine replacement therapy and more intensive postoperative follow-up to monitor healing progression and mitigate complications.

### Limitations

This study has several limitations that require careful consideration. Despite presenting an adequate cohort of patients for systematic review, the relatively small number of studies and patients available for pooled statistics limits the strength of the associations that can be inferred. The small sample sizes in several studies may have reduced the statistical power of our analysis, potentially leading to an inability to detect significant associations where they may exist. We also acknowledge that the use of mixed methods, ranging from prospective to retrospective designs, introduces varying levels of evidence and can introduce biases which may impact the meta-analysis results. The heterogeneity observed across the studies—due to variations in surgical techniques, definitions of smoking status, and patient characteristics—was addressed using a random-effects model, which allowed for more generalized conclusions despite these differences. However, this heterogeneity still complicates the interpretation of the results. Smokers often have other underlying health issues like obesity, diabetes, and cardiovascular disease, which can independently affect HA outcomes and confound the observed associations. Future research should include larger, more diverse cohorts and control for additional confounding variables to better understand the relationship between smoking and HA outcomes.

## Conclusion

Mixed evidence exists regarding smoking’s impact on hip pathology outcomes after HA, with moderate-quality studies and limited data. While some individual studies suggested a potential link between smoking and conversion to THA, our meta-analysis found no statistically significant association. Similarly, no significant association was observed between smoking and RHA. However, smoking was consistently linked to higher rates of adverse outcomes such as VTE, HA failure, increased opioid use, and infection, and smokers were seen to experience increased conversion to THA in general. Clinicians should cautiously plan HA surgery for patients with extensive smoking history, as the risks of this demographic factor have yet to be delineated. Emphasis of perioperative counseling on smoking cessation is critical in reducing postoperative complications profiles for patients with preexisting comorbidity burden. VTE prophylaxis stratification can be considered using the VTEstimator, though the weightage of smoking history as a risk factor must be done on a case-by-case basis with consideration of the patient’s existing comorbidity profile until future research can establish adequate risk weighting for smoking. Thus, future studies should include smoking as a standard demographic variable and utilize extended follow-up periods to better capture long-term outcomes.
